# Emerging Therapeutic Agents for Colorectal Cancer

**DOI:** 10.3390/molecules26247463

**Published:** 2021-12-09

**Authors:** Marianna Nalli, Michela Puxeddu, Giuseppe La Regina, Stefano Gianni, Romano Silvestri

**Affiliations:** 1 Department of Drug Chemistry and Technologies, Sapienza University of Rome, 00185 Rome, Italy; marianna.nalli@uniroma1.it (M.N.); michela.puxeddu@uniroma1.it (M.P.); giuseppe.laregina@uniroma1.it (G.L.R.); 2 Department of Biochemical Sciences “Rossi Fanelli”, Institute of Biology and Molecular Pathology of CNR, Sapienza University of Rome, 00185 Rome, Italy; stefano.gianni@uniroma1.it

**Keywords:** cancer, CRC, emerging targets, anticancer agents

## Abstract

There are promising new therapeutic agents for CRC patients, including novel small-molecule inhibitors and immune checkpoint blockers. We focused on emerging CRC’s therapeutic agents that have shown the potential for progress in clinical practice. This review provides an overview of tyrosine kinase inhibitors targeting VEGF and KIT, BRAF and MEK inhibitors, TLR9 agonist, STAT3 inhibitors, and immune checkpoint blockers (PD1/PDL-1 inhibitors), for which recent advances have been reported. These new agents have the potential to provide benefits to CRC patients with unmet medical needs.

## 1. Introduction

Colorectal cancer (CRC) is a leading cause of cancer mortality worldwide, being the second- and third-most frequently occurring cancer in women and men, respectively [1]. In 2018, CRC was the third-most-common tumor, after lung and breast cancer, with 1.8 million new cases diagnosed (10.6%, except nonmelanoma skin cancer) [2]. The risk of developing CRC is influenced by both environmental and genetic factors, the majority of CRCs being sporadic rather than familial [3]. Most common familial colon cancer syndromes, together accounting for nearly 5% of CRC cases, are familial adenomatous polyposis and Lynch syndrome (hereditary nonpolyposis colorectal cancer) [4,5,6]. Although there may be an increased risk of developing CRC associated with the intake of some food items (i.e., processed meats), the absolute risk is small and only occurs with daily consumption [3]. However, cigarette smoking [4], excessive alcohol consumption [7], long-term androgen deprivation therapy (ADT) with gonadotropin-releasing hormone (GnRH) agonist [8], orchidectomy [9], and cholecystectomy [10] have been associated with increased incidence of and mortality from CRC.

Radiotherapy and/or chemotherapy is the main treatment both in the early stage (stage 0) of non-metastatic CRC and in stages I to III, whereas systemic chemotherapy is generally used for patients with stage IV CRC (metastatic CRC (mCRC)). The 5-year relative survival rate for all cancers combined diagnosed between 2009 and 2015 was 67% overall [11]. Instead, low lung cancer survival rates reflect the large proportion of patients (57%) diagnosed with mCRC for which the 5-year relative survival rate is 5% [12].

## 2. Current Therapies

The *KRAS* (Kirsten rat sarcoma virus) gene was identified more than 25 years ago as a causative agent of oncogenesis [13]. A large proportion of solid tumors, from pancreatic [14] to pulmonary adenocarcinomas [15,16], are characterized by the presence of *KRAS* mutations. *KRAS* is a component of the tyrosine kinase signaling pathways; downregulation of these pathways may underlie much of the therapeutic value of the tyrosine kinase inhibitors (TKIs) [17]. The absence of *KRAS* mutations has been correlated to the clinical response to the monoclonal antibody cetuximab, an inhibitor of the epidermal growth factor receptor (EGFR) [18,19]. *KRAS* mutation testing is important in the selection of protocols anti-EGFR CRC also in other solid tumors [20].

Patients with mCRC are divided in two groups for first-line treatment, depending on the *KRAS* mutational status. The majority of patients with wild-type (WT) *KRAS* typically are treated with an antibody, either the vascular endothelial growth factor (VEGF-A)-targeted antibody bevacizumab or an epidermal growth factor receptor (EGFR)-targeted antibody, cetuximab or panitumumab, and with chemotherapy based on oxaliplatin and/or irinotecan. Patients bearing the *KRAS* mutation generally are treated with bevacizumab and chemotherapy. Both bevacizumab and chemotherapy are commonly used as maintenance therapy, independently of the *KRAS* status. Alternative, chemotherapy regimens with the same drugs are used as second-line treatments. Additional options independent of the *KRAS* mutational status are based on the VEGFR-2 targeting antibody ramucirumab or the VEGF inhibitor aflibercept. Aflibercept has been approved for treatment of mCRC in combination with FOLFIRI, a chemotherapeutic regimen including folic acid, 5-fluorouracil, and irinotecan, for patients whose disease progresses during or after treatment with an oxaliplatin-containing regimen [21]. Ziv-aflibercept is approved for the treatment of mCRC; its molecular structure is the same as that of aflibercept [22]. Patients who do not respond to oxaliplatin and irinotecan chemotherapy are treated as third-line option, with the small-molecule tyrosine kinase inhibitor (TKI) regorafenib or with the oral chemotherapy combination of trifluridine and tipiracil [23]. Potential therapies for CRC have been consistently analyzed in the excellent reviews by Xie [24] and Das [25]. In this work, we do not aim to provide a comprehensive review of all the approaches to CRC treatment but rather to focus on emerging therapeutic agents for CRC that have shown the potential for progress in clinical practice.

## 3. Emerging Therapeutic Agents

### 3.1. Tyrosine Kinase Inhibitors Targeting VEGF

Nintedanib (**1**) (methyl 2-hydroxy-3-[*N*-[4-[methyl-[2-(4-methylpiperazin-1-yl)acetyl]amino]phenyl]-*C*-phenylcarbonimidoyl]-1*H*-indole-6-carboxylate) is a small-molecule TKI that targets platelet-derived growth factor receptors (PDGFRs) α and β, fibroblast growth factor receptors (FGFRs) 1–3, and vascular endothelial growth factor receptors (VEGFRs) 1–3 [26]. In addition, **1** inhibits members of the Src family of tyrosine kinases, including Src, Lck, and Lyn, and fms-like tyrosine kinase 3 (FLT-3) with median inhibitory concentrations (IC_50_ values) < 100 nM [26]. **1** behaves as a competitive inhibitor that blocks intracellular signaling through binding to the ATP-binding pockets of these receptors [27]. Nintedanib has been approved for the treatment of idiopathic pulmonary fibrosis (IPF) in many countries, including Europe and the USA, and the standard dosing in adults is 150 mg twice daily [28,29]. Compound **1** has proved to inhibit the initiation and progression of lung fibrosis, which might have relevance to the treatment of patients with interstitial lung diseases [30].

Compound **1** has been shown to delay or arrest tumor growth in xenograft models of solid tumors [27]. In monotherapy regimen, **1** exhibited an antiangiogenic effect in 67% of patients with advanced, refractory CRC in a phase I study and stabilized the progress of the disease with an acceptable safety profile [31,32,33]. Based on these results, **1** was hypothesized to be a suitable treatment for patients with CRC who had failed all currently approved therapies but could benefit from additional treatment. In a phase III LUME-Colon 1 study, **1** did not improve overall survival but provided a statistically significant but modest increase in progression-free survival in heavily pre-treated patients with mCRC that was refractory to all currently approved standard therapies and was well tolerated [34]. In the LUME-Colon 1 (NCT02149108) phase III study of **1** in advanced CRC, pre-specified biomarker analyses investigated the association of CRC consensus molecular subtypes (CMS) [35] and tumor genomic and circulating biomarkers with clinical outcomes. These studies suggest the potential for greater response to **1** treatment in the unclassified/mixed CMS subgroup, despite these tumors showing heterogeneous patterns of CMS mixtures [36].

Inhibition of angiogenesis via the VEGF pathway has proven to be a valid approach in the treatment of CRC; meanwhile, the drug combination of cytotoxic agents remains the main approach to the treatment of mCRC. Angiogenesis targeting agents have been approved, as monotherapy or in combination with 5-FU-based regimens, achieving an overall survival of nearly 30 months of mCRC patients [37,38].

Regorafenib (**2**) (4-[4-[[4-chloro-3-(trifluoromethyl)phenyl]carbamoylamino]-3-fluorophenoxy]-*N*-methylpyridine-2-carboxamide) is an orally bioavailable small molecule with potential antiangiogenic and antineoplastic activities. It behaves as a multi-TKI with kinase receptors in angiogenic, stromal, and oncogenic tissues. Its antiangiogenic activity is due to the inhibition of VEGFR 2 and VEGFR 3 and Ret, Kit, PDGFR, and Raf kinases [39,40]. Compounds **2** and TAS102, the latter composed of the thymidine phosphorylase inhibitor tipiracil and the cytotoxin trifluridine, have both shown to improve survival in patients who had previously received all available standard therapies; they are approved for use in refractory CRC [41,42]. In addition, **2** was approved by the FDA in 2012 and the EMA in 2013 for the treatment of mCRC. In the CORRECT trial, **2** led to significant improvements in the median overall survival and median progression-free survival [43]. The phase III CONCUR trial evaluated further efficacy and safety of **2**. The results were consistent with the previous CORRECT trial in both median overall survival and median progression-free survival [39,44]. A Japanese study compared the efficacy of **2** and trifluridine/tipiracil in patients with mCRC who are refractory to standard chemotherapy. In this study, significant difference in overall survival between mCRC patients treated with **2** and trifluridine/tipiracil was observed [45]. In a recent phase Ib trial, **2** plus nivolumab, a recombinant, inhibitory anti-idiotypic antibody, showed a manageable safety profile and encouraging antitumor activity for gastric and colorectal cancer [46].

Fruquintinib (**3**), (6-[6,7-dimethoxyquinazolin-4-yloxy]-*N*, 2-dimethylbenzofuran-3-carboxamide) is a small-molecule TKI with high affinity for VEGFRs 1, 2, and 3. It has shown to inhibit tumor angiogenesis in vivo and in vitro [47]. From data analysis of the FRESCO (NCT02314819) trial, fruquintinib showed excellent efficacy and tolerable toxicity in patients with pre-treated mCRC [48,49]. On 2018, **3** was approved by the China Food and Drug Administration (CFDA) to treat patients with mCRC who had undergone at least two unsuccessful standard antitumor therapies, including fluoropyrimidine, oxaliplatin, and irinotecan, with or without prior use of anti-VEGF or anti-epidermal growth factor receptor (EGFR) treatment [50,51]. As a drug, **3** improves both overall survival and progression-free survival in heavily pre-treated mCRC patients and is approved as a third-line treatment for mCRC [44]. Despite the effectiveness exhibited by **3** in mCRC treatment, some issues need to be explored, namely the absence of molecular biomarkers that accurately predict the efficacy [49].

A combination of **3** and anti-PD-1 for the microsatellite-stable phenotype CRC therapy was investigated. After several failed attempts at multiline therapy, the patient treated with a drug combination of **3** and anti-PD-1 achieved a rapid response. In a murine syngeneic model, cotreatment significantly inhibited tumor growth and promoted survival time compared with the single drug. Moreover, such cotreatment decreased angiogenesis, enhanced normalization of the vascular structure, alleviated tumor hypoxia, and proved to reprogram the immune microenvironment. In summary, these studies indicated that a **3** and anti-PD-1 combination could synergistically suppress CRC progression and alter the tumor microenvironment in favor of antitumor immune responses [52].

Over the past decade, several anti-VEGFR TKIs in use as anti-cancer agents have been evaluated for the treatment of mCRC. Among them, axitinib, apatinib, and famitinib, have proven to be effective in the treatment of mCRC.

Axitinib (**4**) (*N*-methyl-2-[[3-[(*E*)-2-pyridin-2-ylethenyl]-1*H*-indazol-6-yl]sulfanyl]benzamide) is a VEGF and PDGF inhibitor exerting an antiangiogenic effect. As an inhibitor of VEGFRs 1, 2, and 3, it is used in the treatment of advanced renal cell carcinoma [53,54]. Compound **4** was evaluated in phase II trials for efficacy and safety as a first-line maintenance therapy for mCRC. In both trials, **4** demonstrated to be an effective agent in the first-line maintenance therapy of mCRC, a promising candidate for the maintenance therapy of mCRC [55,56]. A combination of **4** and navitoclax (ABT263, an antagonist of the Bcl-2 family with selective binding to the apoptosis suppressors Bcl-2 and Bcl-XL) has shown synergistic effects on RAmutant colon cancer cells. This drug combination inhibited the cancer cell growth and enhanced apoptosis. In addition, the combination downregulated slightly AKT and Wnt/β-catenin signaling pathways in *KRAS*-mutant colon cancer cells. This synergistic effect can be useful for therapy in colon cancer cells harboring the *RAS*-mutant and for treating refractory cancers driven by oncogenes, such as *KRAS* [57].

Apatinib (**5**), also known as rivoceranib (*N*-[4-(1-cyanocyclopentyl)phenyl]-2-(pyridin-4-ylmethylamino)pyridine-3-carboxamide), is a small-molecule TKI with antiangiogenic and antineoplastic activities. It inhibits VEGFR 2, decreasing VEGF-stimulated endothelial cell migration and proliferation and tumor microvessel density, and also inhibits c-KIT, RET, and c-SRC [58]. The CFDA approved **5** for the treatment of advanced chemotherapy-refractory gastric cancer [59]. In a phase II trial on a small cohort, **5**, at a daily single dose of 500 mg orally, was effective in the treatment of refractory mCRC in patients who had undergone at least two standard treatments. The dose was reduced to avoid adverse events [60]. Compound **5** has shown to enhance anti-PD-1 antibody therapy for colon cancer in mice by upregulating PD-L1 expression in various colon cancer cells both at the mRNA and protein levels. In addition, **5**-treated cancer cells hampered activation and IFN-γ secretion of T cells in the co-culture system. Treatment with a combination of **5** and anti-PD-1 by intraperitoneal injection showed more significant inhibition of transplanted tumor growth in mice than single-drug treatment. Overall, this study demonstrated the enhancement of anti-PD-1 antitumor efficacy in a syngeneic mouse model (CT-26 cells in Balb/c) by **5** via upregulating PD-L1 expression as well as angiogenesis inhibition and provided a rationale for the combination of **5** and anti-PD-1 antibody for colorectal cancer treatment in the clinic [61]. In addition, **5** alleviated hyperangiogenesis and hypoxia in tumor microenvironment and converted the immunosuppressive tumor microenvironment into an immunostimulatory one in VEGFA-overexpressed tumors [62].

Famitinib (**6**) (5-[2-(diethylamino)ethyl]-2-[(*Z*)-(5-fluoro-2-oxo-1*H*-indol-3-ylidene)methyl]-3-methyl-6,7-dihydro-1*H*-pyrrolo[3,2-c]pyridin-4-one) is a TKI that binds VEGFRs 2 and 3, PDGFR, FLT-1 and FLT-3, and the stem cell factor receptor (c-KIT and SCFR) and is involved in the dysregulation of several solid tumors, such as renal cell carcinoma and nasopharyngeal cancer [63,64]. In a double-blind phase II trial of patients with refractory mCRC, progression-free survival of the group treated with **6** was longer than that of the placebo group and the disease control rate increased; however, the overall survival and the objective response rate did not increase, though **6** showed an acceptable safety profile [65,66].

Surufatinib (Sulfatinib) (**7**) (*N*-[2-(dimethylamino)ethyl]-1-[3-[[4-[(2-methyl-1*H*-indol-5-yl)oxy]pyrimidin-2-yl]amino]phenyl]methanesulfonamide) is a TKI that targets VEGFR 1, 2, and 3; FGFR1; and colony stimulating factor 1 receptor (CSF1R). It has shown dual activity, of anti-angiogenesis and regulating cancer immunity. In December 2020, **7** was approved as a monotherapy for unresectable locally advanced or metastatic, progressive nonfunctioning, well-differentiated (grade 1 or 2) extra pancreatic neuroendocrine tumors (epNETs) in China [67]. In February 2021, clinical trials NCT04653480 (recruiting China) began to determine the efficacy and safety of **7**, toripalimab, and chemotherapy in second-line RAS/BRAF mutant and microsatellite stable (MSS) colorectal cancer. The estimated primary completion date is January 2023 [68].

### 3.2. Tyrosine Kinase Inhibitors Targeting KIT

Masitinib (**8**) (4-[(4-methylpiperazin-1-yl)methyl]-*N*-[4-methyl-3-[(4-pyridin-3-yl-1,3-thiazol-2-yl)amino]phenyl]benzamide) is a TKI that specifically targets KIT, which is overexpressed or mutated in several tumors, and inhibits recombinant PDGFR and the intracellular kinase Lyn [69]. Recently, a drug repurposing screen identified compound **8** as a 3CLpro inhibitor that blocks replication of SARS-CoV-2 in vitro [70].

In several animal and human malignancies, activated c-KIT receptor and tryptase play a key role in tumor angiogenesis [71,72], contributing to tumor cell invasion and metastasis [73]. The increased activation of the c-KITR pathway causes the activation of mast cells and the induction of angiogenic cytokines (such as VEGF, PDGF, FGF-2) (71,72) and tryptase degranulation [74]. Increased mast cell activity in the tumor microenvironment is linked to poor prognosis and a protumoral immune response in CRC [75]. In vitro, **8** acts as a chemosensitizer of 5-FU and the camptothecin irinotecan in CRC cell lines. A combination of **8**, at a reduced dose to minimize the risk of toxicity, with FOLFIRI showed favorable efficacy in mCRC patients (50% with mutated *KRAS*) with an acceptable safety. Therefore, **7** may provide patients an alternate therapy for mCRC [76].

**Figure 1:**
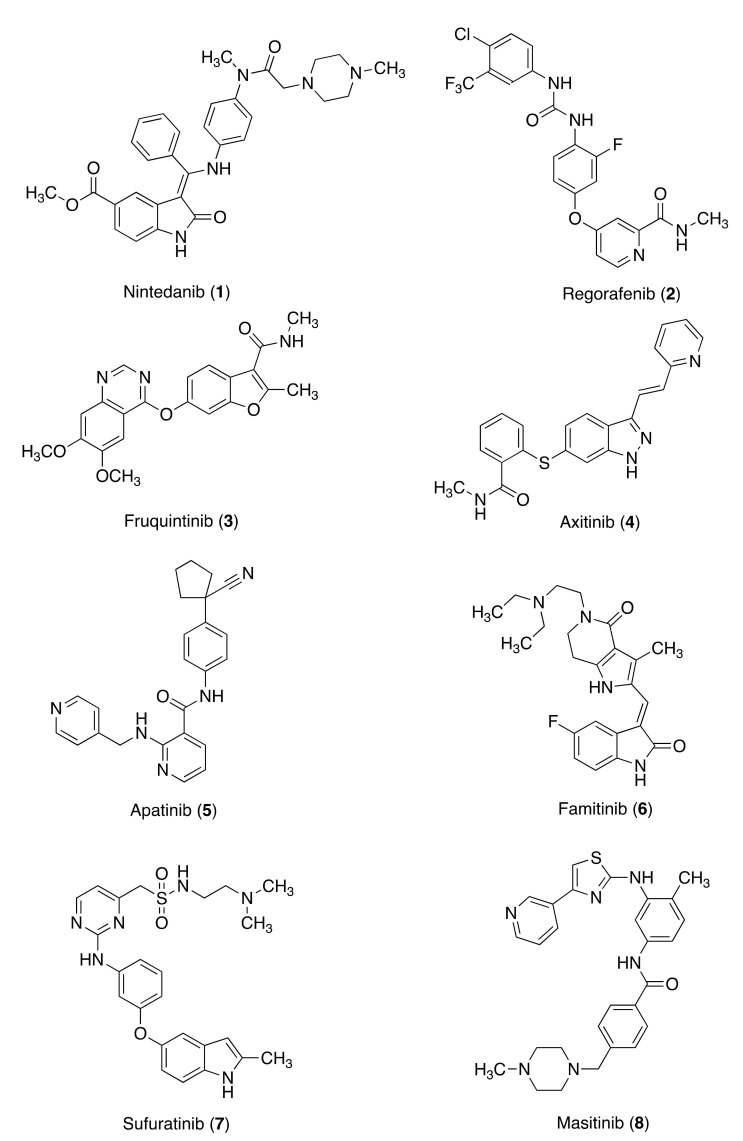
Tyrosine kinase inhibitors targeting VEGF and KIT.

TKIs have been shown to reduce regulatory T cells (Treg) in tumor-bearing animals and patients with metastatic renal carcinomas. Treg were analyzed in the peripheral blood of patients with mCRC treated with anti-VEGR-A antibody bevacizumab and in colon-cancer-bearing mice (CT26) treated with drugs targeting the VEGF/VEGFR axis. In CT26-bearing mice, anti-VEGF antibody and sunitinib treatments was found to reduce Treg, but **8**, a TKI not targeting VEGFR, did not. Anti-VEGF-A treatment decreased Treg proliferation in mice as well as in patients with mCRC. This study led to the conclusion that anti-VEGF-A treatment decreases Treg proliferation in mice as well as in patients with mCRC [77] (Figure 1).

### 3.3. BRAF Inhibitors

BRAF is a serine/threonine enzyme in the RAF/MEK/ERK signaling pathway. Interfering with the RAF/MEK/ERK signaling pathway results in the inhibition of tumor cell growth. The BRAF^V600E^ mutation, a Val-to-Glu mutation at residue 600 (V600E), represents nearly 90% of BRAF gene mutations. It is constitutively activated in the RAF/MEK/ERK signaling pathway and is often upregulated in several human tumors. Up to 15% of CRC patients present mutated BRAF, and such mutations are associated with a poor prognosis. mCRC treatment can be considered in terms of BRAF inhibition following the notably successful outcome of BRAF inhibition in BRAF mutant metastatic melanoma.

Vemurafenib (9) (N-[3-[5-(4-chlorophenyl)-1H-pyrrolo[2,3-b]pyridine-3-carbonyl]-2,4-difluorophenyl]propane-1-sulfonamide) is a selective inhibitor of BRAFV600E-mutated kinase, resulting in the inhibition of tumor cells overexpressing this mutation in the RAF/MEK/ERK signaling pathway. A study of 9 as a single agent in two cohorts of mCRC and metastatic melanoma patients with a tumor harboring a BRAFV600E mutation gave a partial response of 5% with a progression-free survival of 3.7 months [78]. The combination of 9 with the anti-VEGF human antibody panitumumab provided 17% partial responses and was well tolerated [79]. Another phase Ib/II study evaluated 9 in combination with cetuximab and irinotecan. The combination induced a response in 35% of the patients, with a median duration response of 8.8 months and progression-free survival of 7.7 months [80].

**Figure 2:**
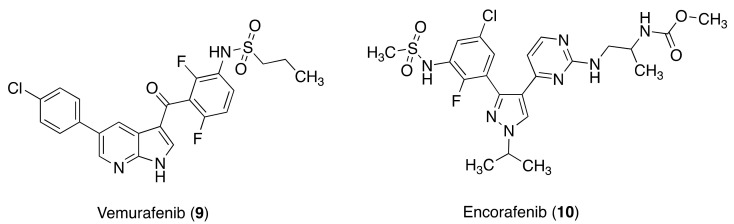
BRAF inhibitors.

Encorafenib (**10**) (methyl *N*-[(2S)-1-[[4-[3-[5-chloro-2-fluoro-3-(methanesulfonamido)phenyl]-1-propan-2-ylpyrazol-4-yl]pyrimidin-2-yl]amino]propan-2-yl]carbamate) is a second-generation inhibitor of BRAF kinase with activity against BRAF, BRAF^V600E^, and CRAF. However, **10** inhibits the RAF/MEK/ERK signaling pathway only in cells harboring the BRAF^V600E^ mutation, with the highest sensitivity observed in BRAF^V600E^ melanoma and CRC cell lines [81]. Early clinical studies of compound **10**, alone or in combination with other drugs, focused mainly on melanoma and CRC. As a monotherapy, **10** was evaluated in refractory mCRC patients with BRAF^V600E^ mutation in study CLGX818X2101, where it showed modest antitumor activity [82]. A phase Ib/II study was launched to evaluate **10** in combination with cetuximab and alpelisib, a PI3K inhibitor, because activation of the PI3K/AKT pathway was hypothesized to cause resistance to BRAF inhibitors [83]. The objective responsa rate in the phase Ib part of this study was 19% in patients who received **10** plus cetuximab and 18% for patients who received triplet therapy with alpelisib. Results of phase II were similar to those observed in the phase Ib part [84]. The study demonstrated that the triple combination achieves the greatest clinical benefit (Figure 2).

### 3.4. MEK Inhibitors

Binimetinib (**11**) (6-(4-bromo-2-fluoroanilino)-7-fluoro-*N*-(2-hydroxyethoxy)-3-methylbenzimidazole-5-carboxamide) is a mitogen-activated inhibitor of protein kinase 1 and 2 (MEK1 and 2). MEK1 and 2 are dual-specificity Thr/Tyr kinases involved in the activation of the RAS/RAF/MEK/ERK signaling pathway. Compound **11** was approved by the FDA for the treatment of patients with unresectable or metastatic melanoma with a BRAF^V600E^ or BRAF^V600K^ mutation in combination with **10**. In the BEACON trial, **11** was evaluated in combination with **10** and cetuximab for the treatment of patients with BRAF^V600E^-mutated mCRC. Treatment with **10** and cetuximab, with or without **11**, has shown impressive improvements in clinical outcomes in the context of tolerable toxicity, compared with standard chemotherapy (87). In the phase Ib/II study CMEK162X2116 (NCT01927341), **11** was evaluated in combination with the anti-EGFR panitumumab in mCRC patients. Compounds **11** with **10** combined in a dual or triple regimen, were investigated in three clinical studies in patients with a variety of tumor types harboring a BRAF^V600^ mutation [85,86]. A phase Ib/II study (NCT01543698) of **10** in combination with **11** in adult patients with BRAF-dependent advanced solid tumors has estimated the completion date of 31 July 2021 [87].

Cobimetinib (**12**) ([3,4-difluoro-2-(2-fluoro-4-iodoanilino)phenyl]-[3-hydroxy-3-[(2S)-piperidin-2-yl]azetidin-1-yl]methanone) is a specific inhibitor of MEK1 resulting in the inhibition of phosphorylation, the extracellular signal-related kinase 2 (ERK2). Compound **12** inhibits cell proliferation in addition to inducing G1 phase arrest and apoptosis in HCT116 colorectal cancer cells, suggesting that **12** may be useful in colorectal cancer therapy. These studies showed that some key genes of DNA replication highly expressed in colorectal cancer tissues were downregulated by **12** in HCT116 cells and genes with low expression in colorectal cancer tissues were upregulated by **12**, including PRKCA, PI3K, RTK, and PKC. After **12** treatment, the inhibition of the PKC and PI3K pathways can increase its cytotoxicity in HCT116 cells. Moreover, **12** has shown to enhance the efficacy of 5-FU by decreasing thymidylate synthetase, an important direct target of 5-FU [88].

A study was conducted to determine whether the CDK1 antagonist enhances the efficacy of MEK inhibition in BRAF^V600E^ colorectal cancer cells. These cancer cells expressing CDK1 were stimulated to apoptosis upon siRNA knockdown or the knockdown of small molecules, such as the CDK1 inhibitor RO-3306 or the CDK1,2,5,9 inhibitor dinaciclib. A combination of RO-3306 or dinaciclib with MEK inhibitor **12** showed synergistic induction of apoptosis and reduced cell proliferation compared to monotherapy. This study established the therapeutic effectiveness of the treatment of BRAF^V600E^ colorectal cancers based on the dual CDK1 and MEK inhibition mechanism [89].

Selumetinib (**13**) (6-(4-bromo-2-chloroanilino)-7-fluoro-*N*-(2-hydroxyethoxy)-3-methylbenzimidazole-5-carboxamide) is the chloro analogue of **11**. A combination of 5-FU and an MEK inhibitor in sequence has shown synergistic effects in *KRAS* or BRAF mutant colon cancer models, compared to monotherapy. Moreover, the administration of 5-FU followed by **13** showed superior synergy compared with the reverse or concomitant treatment in six cell lines [90].

**Figure 3:**
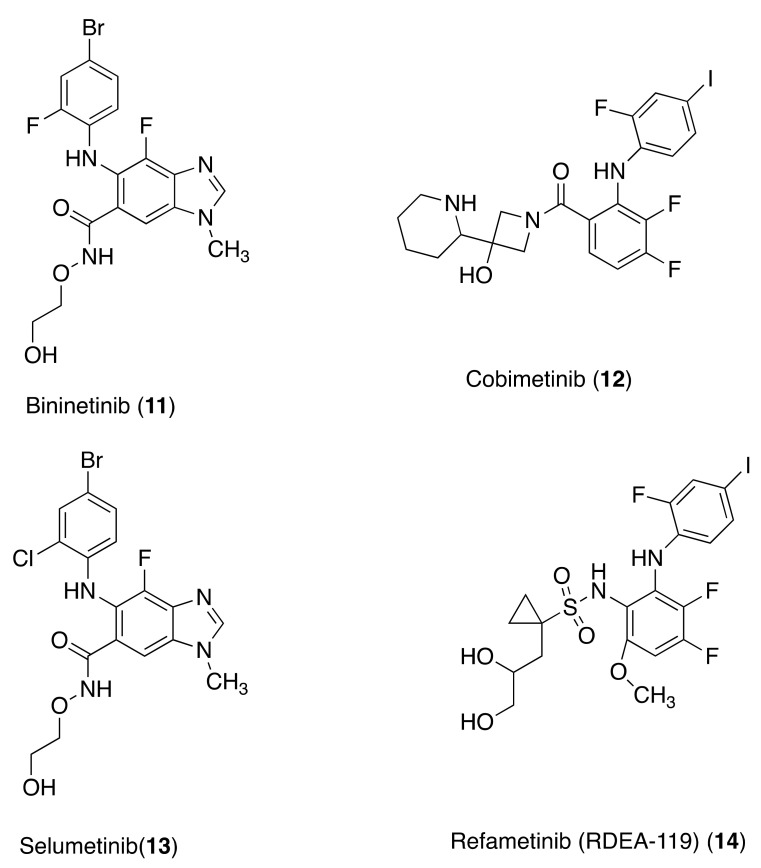
MEK inhibitors.

Refametinib (RDEA-119) (**14**) (*N*-[3,4-difluoro-2-(2-fluoro-4-iodoanilino)-6-methoxyphenyl]-1-[(2*S*)-2,3-dihydroxypropyl]cyclopropane-1-sulfonamide) is an inhibitor of MAP2K1, a dual-specificity Thr/Tyr kinase involved in the RAS/RAF/MEK/ERK signaling pathway. Studies aimed to identify new therapeutic agents and elucidate the possible mechanism(s) of resistance to anti-HER2 treatments were performed using LIM1215 and SW48 human colon cancer cell lines and the corresponding HER2-amplified derivatives and different xenograft models [91]. To validate the potential efficacy of these agents, tumor xenografts with HER2 gene amplification derived from human mCRC patients (94) were used. The combined treatment with **14** and pictilisib, a small-molecule class I PI3K inhibitor, was more effective than treatment with a combination of the EGFR inhibitor lapatinib and trastuzumab in parental SW48 and LIM1215 and in HER2-amplified human colon cancer cell lines. The combined treatment with **14** and pictilisib suppressed almost completely LIM1215-HER2 and SW48-HER2 tumor growth after 4-week treatment in mice injected with LIM1215-HER2 and SW48-HER2 cells [92]. Collectively, the results of these studies show that the combined inhibition of MEK and PI3KCA causes a significant reduction in tumor growth in several different models of HER2-amplified CRC both in vitro and in vivo [92]. Another study, using **14** as an MEK inhibitor, concluded that combined treatment with MEK, EGFR, and PD-L1 inhibitors may be a promising treatment option for a particular subtype of CRC patients [93] (Figure 3).

### 3.5. TLR9 Agonist

Toll-like receptor type 9 (TLR9) is being investigated as a therapeutic target for the development of infective and anticancer agents, vaccine adjuvants, and antiallergens [94]. TLR9 activation triggers a signaling cascade involving NF-kB, IFN-α and -γ, NK cells, and immunomodulatory functions of myeloid dendritic cells (mDCs) to initiate adaptive immune responses [95]. TLR9 ligands are phosphorothioate oligonucleotides including short linear DNA molecules linked by phosphothioester bonds in the DNA backbone to alter the chemical properties of native-state DNA. On the basis of structure and biologic profile, TLR9 ligands have been grouped into the classes, A, B, and C. Lefitolimod (**15**), known as double-Stem Loop ImmunoModulator (dSLIM), showed a covalently close dumb-bell-shaped structure without any unnatural modification. The structure of **15** reported in Ref. [96] is made by two single-stranded loops of 30 nucleotides each. The two loops are spanned and spaced by a double-stranded stem of 28 base pairs [96]. **15** has been evaluated for treatment in individuals with HIV infection to investigate whether it could reactivate latent HIV, improve immune responses in participants with HIV, reduce the size of the latent HIV reservoir, and delay the time to a viral rebound after the interruption of ART [97] (NCT02443935 [98]). Compound **15** has been evaluated in a phase II clinical trial in patients with advanced colorectal carcinoma (IMPACT, NCT01208194). This phase II study was conducted in patients after first-line standard chemotherapy regimens with fluoropyrimidines/leucovorin and irinotecan or oxaliplatin combined with a standard dose of bevacizumab. Completely chemotherapy-free intervals were applicable in patients with advanced CRC who achieved disease control after initial first-line chemotherapy [99]. Based on the results of the IMPACT study, **15** was evaluated in a phase III clinical trial (NCT02077868) as a maintenance therapy in mCRC patients who had responded to first-line therapy. It was found that **15** is not superior to the standard of care as a single-agent maintenance treatment in patients with mCRC. The study confirmed its favorable safety and tolerability profile [100].

### 3.6. STAT3 Inhibitors

A signal transducer and activator of transcription factor 3 (STAT3) is found to be constitutively active in many cancers, including colon cancer [101], and plays a major role in cancer progression. Napabucasin (**16**) (2-acetylbenzo[f][1]benzofuran-4,9-dione) is a small-molecule inhibitor of STAT3 that is activated by NAD(P)H:quinone oxidoreductase 1, an antioxidant enzyme overexpressed in many solid tumors [102]. In a phase III trial, **16** monotherapy was unable to improve survival among mCRC patients compared with placebo [103]. Another phase III trial, the CanStem303C trial (NCT02753127), of **16** in combination with the standard chemotherapy in patients with mCRC is ongoing, based on promising results obtained in a phase Ib/II clinical study [104]. A phase I/II trial was conducted to assess the efficacy and safety of **15** plus pembrolizumab, a humanized antibody used in cancer immunotherapy in mCRC patients. This combination showed antitumor activity and safety profile similar to those of **15** monotherapy or pembrolizumab monotherapy in patients with microsatellite-stable mCRC as well as microsatellite-instable high mCRC. Four patients (among a small population) with microsatellite-stable mCRC MSS responded to this combination in association with tumor biomarkers [105].

Stattic (**17**) (6-nitro-1-benzothiophene 1,1-dioxide) is a small molecule that selectively inhibits STAT3 by blocking the function of its SH2 domain [106]. A study reported that the suppression of STAT3 activity significantly enhances the efficacy of doxorubicin in the induction of immunogenic cell death in CT26 colon cancer cells. The treatment of CT26 cells with **17** in combination with doxorubicin results in synergistic antitumor effects. Moreover, the treatment of these cancer cell lines with a combination of **17** and doxorubicin induces dendritic cells functional maturation and IL-12 secretion [107].

**Figure 4:**
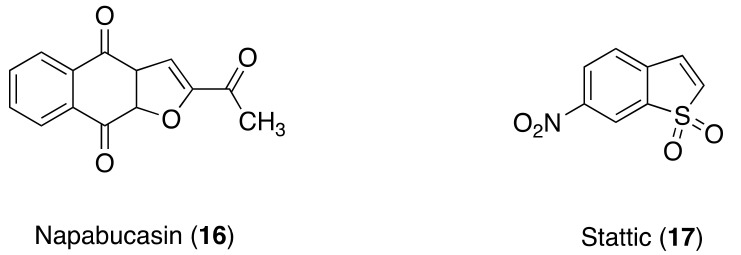
STAT3 inhibitors.

Laminin 521 (LN521) protein has shown to promote CRC cell self-renewal and invasion. High levels of laminin alpha 5 were detected in 7/10 liver metastases tissue sections collected from CRC patients, which is a frequent feature of metastatic dissemination in CRC. Exposure of CRC cells to LN521 enhanced STAT3 phosphorylation; incubation with STAT3 inhibitors **16** and **17** was sufficient to block the LN521-driven self-renewal increase [108] (Figure 4).

### 3.7. PD1/PDL-1 Inhibitors

The expression of the PD ligand PDL-1 has been detected in a variety of malignancies and is often associated with a poor prognosis. The PDL-1 ligand binds the programmed cell death 1 (PD-1) receptor expressed on the surface of T cells and other immune cells, thereby inhibiting the activation of T cells. Interfering with the PD-1/PD-L1 pathway leads to a restoration of T cell function and improvement in the anti-tumor immune response [109]. Anti-PD-L1 antibodies play an important role in oncology since clinical trials have shown their impressive efficacy in the treatment of a variety of unresectable tumors [110]. Unfortunately, some patients show an innate resistance to the inhibition of the PD-1/PD-L1 pathway [111] and others develop resistance after an initial response [112]. For this reason, new approaches, with conventional agents or antibodies, are currently being researched to improve the antitumor therapy [113,114]. An exploratory study was conducted to evaluate various combination regimens of chemotherapies with immune checkpoint blockers anti-PD-1 and anti-PD-L1 in several murine syngeneic preclinical models. Capecitabine and oxaliplatin were chosen for murine colon cancer MC38 cells because of similarity to the tumor type in patients [115]. A combination of immune checkpoint inhibitors, such as anti-PD-1 or anti-PD-L1 antibodies, proved to be more effective than either regimen used separately for the combination of capecitabine/oxaliplatin with anti-PD-1 antibody in the MC38 colorectal cancer model.

Strong PD-L1 expression was observed in 37% of mismatch repair (MMR)-proficient and in 29% of MMR-deficient CRC cases [116]. A phase Ib study that combined the anti-PDL-1 atezolizumab with FOLFOX/bevacizumab as the first-line treatment of mCRC, independent of the microsatellite status, showed an interesting outcome, without safety concerns. Phase III trials based on FOLFOXIRI plus bevacizumab (and optionally atezolizumab) identified these as a therapeutic option in selected mCRC patients [117]. A phase II randomized BACCI study demonstrated weak efficacy in delaying tumor progression when adding atezolizumab to capecitabine and bevacizumab for pre-treated patients with mCRC unselected for the microsatellite status [118]. The AtezoTRIBE study (NCT03721653) aimed to assess whether the addition of atezolizumab to an intensified chemotherapy plus bevacizumab might be an effective upfront strategy for the treatment of mCRC, irrespective of the microsatellite status [119].

The anti-PD-1 antibody pembrolizumab was evaluated in the multicohort KEYNOTE-028 (NCT02054806) trial in 20 PD-L1-positive advanced solid tumors. Pembrolizumab demonstrated a favorable safety profile in advanced PD-L1-positive CRC. Antitumor activity was observed in a single patient with microsatellite instability-high CRC [120].

A case of a Lynch syndrome patient with mCRC and urothelial cancer was treated sequentially with anti-PD-1 antibody, with atezolizumab (anti-PD-L1), briefly with pembrolizumab, and finally with the combination of ipilimumab (targeting CTLA-4) and nivolumab (targeting PD1). The patient experienced prolonged disease control with each different regimen the first time it was given over 28 months. This case suggested that some patients with advanced MMR-deficient CRC may experience positive benefits from multiple sequential immune checkpoint blockade regimens [121].

### 3.8. PDZ Domains Inhibitors

An interesting emerging strategy to treat CRC is to target protein–protein interaction domains that are overexpressed in cancer [122,123,124,125]. In fact, whilst these classes of molecules do not possess any enzymatic activity, they typically act at the head of signaling networks, thereby orchestrating multiple pathways. Thus, inhibiting these domains may result in the effective inhibition of multiple enzymes, as their activity could be prevented simultaneously.

PDZ domains are the most abundant protein–protein interaction modules of the human proteome, comprising 266 different domains contained in 150 proteins [126] and characterized by a conserved structural architecture of 80–100 amino acids [127]. The structural architecture of PDZ domains is characterized by a well-defined binding site, which recognizes short sequences typically located at the C-termini of the physiological ligand [128]. The aberrant expression of several PDZ-domain-containing proteins has been associated with different type of cancers, including CRC [129,130,131,132].

From this perspective, a successful example of a promising class of therapeutic agents has been recently described in the context of the inhibition of the PDZ containing protein NHERF1 (Na^+^/H^+^ exchanger 3 regulating factor 1) [133,134]. Previous studies have shown that the oncogenic activities of NHERF1 are correlated to an aberrant subcellular localization. In fact, in the case of CRC, NHERF1 is overexpressed in the cytoplasm and nuclei, where it enhances a cytoprotective autophagic response, elicited by the therapeutic inhibition of the Wnt/β-Catenin signaling cascade. By using a synergy between molecular dynamics and chemical synthesis, several small molecules were designed and tested against NHERF1 [133,134]. The ability of these molecules to bind specifically the PDZ domain of NHERF1 was also monitored by equilibrium fluorescence titrations, allowing an estimation of the observed K_I_. Benchmarking these studies with cellular experiments in cellula will allow lead compounds that represent promising targets to be optimized in future work.

### 3.9. Cancer Stem Cells

Cancer stem cells (CSCs) account for a small population of cells but are involved in the self-renewal and differentiation of several types of cancer, from initiation to recurrence and metastasis [135,136]. Radiation or chemotherapeutic agents may inhibit non-CSCs in a tumor mass but have limited efficacy with CSCs. Accordingly, colorectal CSCs are not sensitive to treatment with 5-FU and oxaliplatin, leading to the recurrence of tumor after chemotherapy and metastasis [137]. The mechanisms behind the colorectal CSC resistance to radiation and chemotherapy remain unclear [138]. Colorectal CSCs share similarities to stem cells from other solid tumors, including self-renewal; differentiation; and persistent activation of multiple signal transduction pathways, such as Wnt/β-catenin, Notch, TGF-β, and Hedgehog [139,140]. Among them, the Wnt/β-catenin pathway plays an important role in stemness maintenance and drug resistance of colorectal CSCs [141].

A group of surface markers allows the identification of colorectal CSC; well-documented markers are CD44, CD133, CD166, Lgr5, ALDH1, and EpCAM [142]. CD44 is a transmembrane glycoprotein that behaves as a (co)receptor for hyaluronic acid, growth factors, and cytokines and is involved in the proliferation of cancer cells [143,144]. CD44 plays a pivotal role in regulating CSC stemness properties, and, along with the CD44v isoform, has been suggested as a biomarker and a therapeutic cancer target [145,146]. In colon cancer, CD44 has been shown to regulate the in vitro and in vivo growth of xenografts in animals [147]. CD44 has also been implicated in the self-renewal and maintenance of pluripotency [148].

Various molecules, including some drugs, have shown to modulate CD44. ICG-001 (**18**) was identified as a CREB-binding protein/catenin antagonist and Wnt modulator that could inhibit the growth of EBV-positive NPC cells through downregulation of the tumor suppressor/pro-differentiator micro-RNA-145 [147]. In addition, **18** downregulates the expression of CD44 and this effect is accompanied by the restored expression of micro-RNA-150 in various nasopharyngeal carcinoma cell lines. Previous studies have demonstrated that the expression of micro-RNA-150 is significantly reduced in various tumor types, including colon cancer [149].

Growth factor receptor bound protein 2-associated protein 2 (Gab2) expression via miR-200c downregulation can promote epithelial-to-mesenchymal transition and CSC-like properties of ovarian cancer cells and enhance the metastatic growth of ovarian cancer in the xenograft model. Thus, miR-200c mimic or CD44 targeted therapy should improve the treatment of ovarian cancer with Gab2 overexpression after the failure of standard therapies [150].

Metformin (**19**), a drug in use for the treatment of diabetes, in combination doxorubicin inhibits both cancer stem cells and non-stem cancer cells in culture. It downregulates the CSC marker CD44 in primary oral cancer cells [151]. Some micro-RNAs, for example, micro-RNA-145 [152], -3129 [153], -200c [150], -143, [154], -328 [155], and -373 [156], were found to be negative modulators of CD44 expression.

Salinomycin (**20a**), an antibiotic isolated from Streptomyces albus, has demonstrated to inhibit CSCs in various types of human cancers, most likely by interfering with ABC drug transporters, the Wnt/β-catenin signaling pathway, and other CSC pathways. A derivative of salinomycin **20b** bearing a cyclopropylamine at position C20 inhibited at nanomolar concentrations HMLER CD24-low/CD44-high cells. In addition, **20** exhibited high selectivity against CSCs and the potential for drug development to prevent cancer resistance [157].

Nonsteroidal anti-inflammatory drug celecoxib (**21**), a COX2 inhibitor, in coordination with the Hsp90 inhibitor 17-allylamino-17-demethoxygeldanamycin might synergistically potentiate the eradication effects on CSCs with CD44 overexpression [158]. Antibodies (mAbs) to CD44 are being investigated for cancer therapy [159], for example, mAb U36 specific to CD44v6 in HNSCC [160] and mAb VFF18 specific to CD44v6 in human squamous cell carcinomas [161].

Galectin-3 (Gal3) is a novel protein that mediates resistance to tumor-necrosis-factor-related apoptosis-inducing ligand (TRAIL), and thus Gal3-positive colorectal CSCs are resistant to chemotherapy regimens, such as FOLFOX (5-FU, oxaliplatin, and leucovorin) and FOLFIRI (5-FU, irinotecan, and leucovorin) [162].

PER3 is a protein that is negatively responsible of colorectal CSC drug resistance and inhibits β-catenin expression and thus restores the sensitivity of CSCs to 5-FU. Interleukin-6 (IL-6) induces resistance to 5-FU through the activation of the Notch-3 signaling pathway [163]. Anti-IL-6 agents can reduce the expression of Oct-4, Klf4, Bmi-1, Lgr5, and Notch-3 and increase cell sensitivity to anticancer drugs [3].

Epigallocatechin-3-gallate (**22**) has been shown to suppress CSC growth in various cancers, but whether it can specifically target CSCs and subsequently sensitize chemoresistant CRC cells to the chemotherapeutic agents remains unknown. In addition, **22** suppressed Notch1, Bmi1, Suz12, and Ezh2 and upregulated self-renewal suppressive-miRNAs, miR-34a, miR-145, and miR-200c, which are some of the key pathways targeted in 5-FU resistant CRC cells [164].

Ipafricept is a recombinant fusion protein that blocks Wnt signaling through the binding of Wnt ligands. In patient-derived ovarian cancer xenografts, ipafricept has shown to decrease the frequency of stem cells, suppress tumor formation, and promote differentiation and shows synergy with taxanes in 2- or 3-day cancer pretreatment [165].

PRI-724 (**23**) is an antagonist of the interaction between β-catenin and its transcriptional coactivator CREB-binding protein. In preclinical studies, **23** promotes differentiation of chemotherapy-insensitive cancer stem cells and tumor-initiating cells, inhibits stroma formation, and decreases metastatic potential [165,166].

In cancer therapy, γ-secretase inhibitors are of interest for investigating their effect on various CSCs, mainly by the modulation of the Notch signaling pathway. MK-0752 (**24**) is a γ-secretase inhibitor that is being investigated in combination with ridaforolimus (MK-8669) in a phase 1 trial of patients with advanced and refractory pancreatic and colorectal cancer [165].

**Figure 5:**
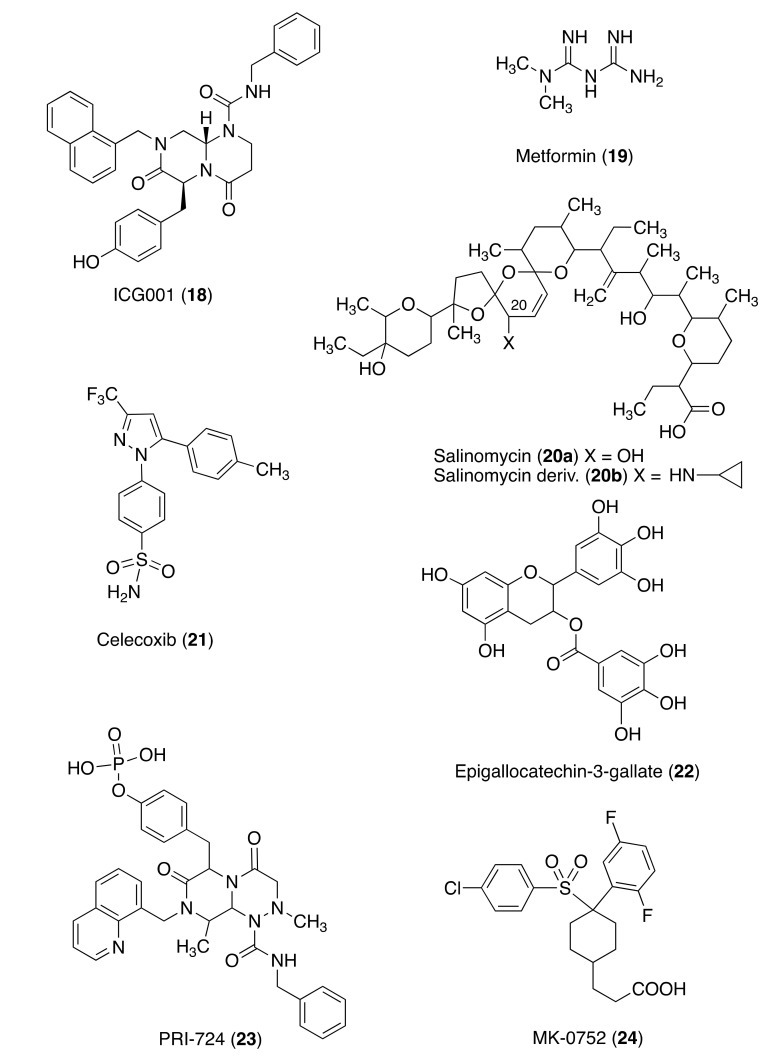
CSC Inhibitors.

Colon CRCs are resistant to radiotherapy due to the upregulation of anti-apoptotic proteins, enhanced DNA damage repair, and dormant/slow cell cycle kinetics [167]. Inhibition of NF-κB signaling reduces radiation-induced stemness; inactivation of Notch signaling inhibits epithelial mesenchymal transition via downregulation of Snail and thus restores sensitivity to radiotherapy [3] (Figure 5).

### 3.10. Chimeric Antigen Receptor (CAR)-T Cells

Chimeric antigen receptor (CAR)-T cells are engineered T cells bearing an artificial receptor that specifically targets tumor-associated antigens (TAAs) of cancer cells. CAR-T cell therapy has shown more efficiency in cancer treatment, particularly regarding blood cancers. CD171 (L1 cell adhesion 20 molecule) is a transmembrane glycoprotein that regulates cell adhesion, survival, growth, migration, and invasion. CD171 has been suggested as a prominent CSC marker in glioblastoma; however, it is also overexpressed in some cancers, such as colon, ovarian, and pancreatic cancer [168]. LGR5 overexpression has been associated with cancer recurrence and tumor formation in breast cancer cells through WNT/β-catenin signaling activation. An anti-LGR5 antibody-drug conjugate inhibited tumor growth and tumor regression in two LGR5-positive xenograft models, LoVoX1.1 (colon cell line) and D5124 (primary human pancreatic) [169]. In another study, an anti-LGR5 antibody-drug conjugate effectively eliminated tumors and prevented those from recurring in a human colon cancer xenograft [170]. EpCAM is more widely expressed on CSCs, including colon cancer, and it is also regarded as a tumor-associated antigen (TAA). CAR-T cells were developed to target CSC-antigen EpCAM to eliminate prostate cancer, demonstrating that EpCAM-specific CAR-T cells have therapeutic potential for cancer treatment [171]. Third-generation EpCAM CAR-T cells were produced to identify the specificity of EpCAM to CRC cells. Infusion with these CAR-T cells significantly restrained tumor growth and development in xenograft mice models [172]. NKG2D-based CAR-T cell therapy was cytotoxic against CRC cells in a dose-dependent manner and significantly inhibited cancer cell growth and prolonged overall survival of mice [173]. HER2-targeted CAR-T cells showed efficacy against HER2^+^ tumors, including tumor regression or even elimination of CRC xenograft and protection against relapse, achieving improved survival benefit [174]. CEA is an acid glycoprotein currently considered a common tumor marker in CRC. In a phase I trial, CAR-T cells were applied in 10 CEA-positive CRC patients: seven were stable following treatment with CAR-T cells, two remained stable for more than 30 weeks, and one even showed tumor regression [175]. CAR-T cells engineered with doublecortin-like kinase 1(DCLK1)-scFv (CBT-511) blocked the growth of subcutaneous xenograft tumors derived from LoVo CRC cells [176]. CAR-T therapy has demonstrated promising potential in some mice models. However, full efficiency of CAR-T cells has been hampered by the chemical and physical tumor environment and the inherent inhibitory mechanisms of T cells [177,178]. Benefits of CAR-T cell therapy appear clearly; however, problems of tumor specificity, toxicity, and lack of flexibility toward improvement still remain and limit their use in patients [179].

### 3.11. Cancer Vaccines

Anticancer vaccines, in particular DC vaccines, have opened up new avenues for cancer therapy [180]. DC vaccines enhance the immunostimulant and immunomodulatory immune system activity aimed at the eradication of cancer cells in the body. Some types of antigens located on the surface of cancer cells can induce a specific immune response. DCs activate an antitumor immune response upon migration to the lymph nodes, where the captured antigens on MHC-I and MHC-II molecules are exhibited to T-lymphocytes [181]. Generally, DC vaccines are prepared from dendric cell or their precursors taken from the blood, followed by maturation and activation steps [182].

Little or no activity has been observed with anti-PD1 therapy in patients with mismatch repair proficiency (MMR-p), which accounts for 96% of advanced CRC cases [183]. The GVAX colon vaccine is an allogeneic, whole-cell, granulocyte-macrophage colony-stimulating factor (GM-CSF)-secreting cellular immunotherapy that induces T cell immunity against a broad range of colon-cancer-associated antigens [184]. The GM-CSF-secreting allogeneic vaccines can increase tumor infiltrating CD8^+^ T effector cells, with the concomitant production of interferon gamma and upregulation of the PD-1/PDL1 pathway [185]. A phase 2 study of GVAX and cyclophosphamide in combination with the anti-PD1 therapy pembrolizumab was conducted in patients with MMR-p metastatic CRC. None of the first 17 patients showed an acceptable radiographic response to therapy, and the trial failed to meet its primary efficacy objective. However, multiple subjects showed a significant decline in their tumor marker levels with study therapy that showed potential for modulating the tumor microenvironment [184].

Many CRC-associated immunogenic proteins are nonmutated and overexpressed in the tumor. Patients with CRC can develop significant immune responses to nonmutated proteins that are important in driving the biology of the disease. Multiepitope vaccines designed to elicit tumor-specific CD4^+^ T cells have potent anti-tumor activity. Vaccines targeting colon-cancer-associated antigens can have prophylactic efficacy in spontaneous intestinal tumor models [186].

A single-antigen vaccine targeting MUC-1 has progressed to clinical evaluation in patients with previous high-risk adenomas as a first attempt in colorectal cancer immuno-prevention [187]. The vaccine was found to be immunogenic, generating high levels of MUC-1-specific antibodies, and safe, with few reported adverse effects. Studies in transgenic mouse models have shown that multi-antigen vaccines are significantly more effective in inhibiting the progression of preinvasive to invasive cancer and preventing clinical disease than single-antigen vaccines alone [188].

COX-2 is overexpressed in a majority of colon cancers, and overexpression is also linked with a poor prognosis [189]. However, the CDC25B protein phosphatase regulates the cell cycle by activating cyclin-dependent protein kinases and could be an early alteration in colorectal cancer pathogenesis [190]. T cell epitopes suitable for inclusion in a vaccine were identified, and a vaccine with antigens CDC25B and COX2 consistently reduced tumor development in two mouse models [186].

## 4. Conclusions

Great efforts are underway to ameliorate the efficacy and tolerability of the therapeutic armamentarium for CRC. Thanks to the rapid progress in pathological and immunological findings, several therapeutic approaches are being developed [23,24]. Novel therapeutic approaches are needed since there are no general regimens that can provide a successful outcome in every treated CRC patient; moreover, current and acquired drug resistance can add further difficulty. Beyond current therapies for CRC patients, there are promising new therapeutic agents, including novel small-molecule inhibitors and immune checkpoint blockers (Table 1). These agents may provide many choices for the treatment of CRC patients suffering from unmet medical needs, promote longer survival, and give hope for a speedy recovery.

## Figures and Tables

**Table 1:** Emerging therapeutic agents for colorectal cancer covered by this review.

Category	Compound	Targets	Disease Indications	Development Phase
TKI targeting VEGF	Nintenabid	VEGF 1–3, PDGF a and b, FGF 1–3, Src family TKs, FLT-3	Idiopathic pulmonary fibrosis, lung fibrosis	Phase III LUME-Colon 1 NCT02149108
	Regorafenib	VEGF 2 and 3; PDGFR; Ret, Kit, and Raf kinases	Refractory CRC, mCRC	Phase III CORRECT, phase III CONCUR Ib phase
	Fruquintinib (also combined with anti-PD-1)	VEGF 1–3	mCRC	Phase III FRESCO NCT02314819
	Axitinib (also combined with navitoclax)	VEGF 1–3, PDGF	Advanced renal cell carcinoma, mCRC	Phase II trial
	Apatinib (also combined with anti-PD-1)	VEGF 2, c-KIT, RET, c-SRC	Advanced refractory gastric cancer, mCRC	Phase II trial
	Famitinib	VEGFRs 2 and 3, PDGFR, FLT-1, FLT-3, c-KIT, SCFR	Renal carcinoma nasopharyngeal cancer, mCRC	Phase II trial
	Surufatinib	VEGFRs 2 and 3, CSF1	Extra pancreatic neuroendocrine tumors, MSS CRC	Phase II NCT04653480
TKI targeting KIT	Masitinib (also combined with FOLFIRI)	KIT	CRC, mCRC	
BRAF	Vemurafenib (also combined with mAb and irinotecan)	BRAF^V600E^	Metastatic melanoma, mCRC	Phase Ib/II
	Encorafenib (also combined with mAb and alpelisib)	BRAF BRAF^V600E^	Melanoma, CRC	Phase Ib/II
MEK	Binimetinib	MEK1 and 2	Metastatic melanoma, mCRC, tumor types harboring a BRAF^V600^ mutation	Phase III BEACON NCT02928224 Phase Ib/II CMEK162X2116 NCT01927341 NCT01543698
	Cobimetinib (also combined with 5-FU or CDK inhibitors)	MEK1	CRC	
	Selumetinib (also combined with 5-FU)	MEK	CRC	
	Refametinib (also combined with pictilisib)	MAP2K1	CRC	
TLR9	Lefitolimod (also combined with ipilimumab)	TLR9 agonist	Latent HIV CRC mCRC	Phase III IMPACT, NCT01208194 NCT02077868
STAT3	Napabucasin (also combined with standard chemotherapy or pembrolizumab)	STAT3	mCRC MSS mCRC MSI mCRC	Phase III CanStem303C t(NCT02753127 Phase I/II
	Stattic (also combined with doxorubicin)	STAT3	CRC	
	Laminin 521 (also combined with stattic)	STAT3	mCRC	
PD1/PDL-1	Atezolizumab (also combined with FOLFIRI and bevacizumab)	PD-L1 inhibitor	mCRC	Phase II BACCI AtezoTRIBE NCT03721653
	Pembrolizumab (also combined with ipilumanb and nivolumband)	Anti-PD-1 antibody	Advanced solid tumors mCRC	Phase 1b KEYNOTE-028 NCT02054806
CSC	ICG-001	CREB catenin antagonist, Wnt modulator	Nasopharyngeal carcinoma CRC	
	Gab2 inhibitor	miR-200c mimic CD44	Ovarian cancer CSC	
	Metformin combined with doxorubicin	CD44	Oral cancer CSC	
	Salynomycin and cyclopropylamine derivative at C20	ABC drug transporter, Wnt/β-catenin, other CSC pathway	Various types of human cancers	
	Celecoxib with Hsp90 inhibitor	CD44	CSC with CD44-overexpression	
	Gal3	TRAIL	Colorectal CSC resistant to FOLFOX and FOLFIRI	
	PER3 protein	β-Catenin	CSC resistant to 5-FU	
	Epigallocatechin-3-gallate	Notch1, Bmi1, Suz12, and Ezh2; miRNAs	CSC, various cancers	
	Ipafricept	Wnt signaling pathway	CSC of ovarian cancer	
	PRI-724	β-catenin–CREB interaction	Chemotherapy-insensitive CSC	
	MK-0752	γ-Secretase	Various CSCs	

## Data Availability

Not applicable.
